# Immune and central nervous system-related miRNAs expression profiling in monocytes of multiple sclerosis patients

**DOI:** 10.1038/s41598-020-63282-3

**Published:** 2020-04-09

**Authors:** Antonella Amoruso, Maria Blonda, Maira Gironi, Roberta Grasso, Valeria Di Francescantonio, Federica Scaroni, Roberto Furlan, Claudia Verderio, Carlo Avolio

**Affiliations:** 1 Department of Medical and Surgical Sciences, University of Foggia, Foggia, Italy; 2 Institute of Experimental Neurology, Division of Neuroscience, San Raffaele Scientific Institute, Milan, Italy; 3 Synlab CAM, Monza, Italy; 4 CNR Institute of Neuroscience, Milan, Italy

**Keywords:** Immunology, Molecular biology, Biomarkers, Diseases, Neurology

## Abstract

It is widely recognized that monocytes-macrophages adopt a wide variety of phenotypes, influencing the inflammatory activity and demyelination in Multiple Sclerosis (MS). However, how the phenotype of human monocytes evolves in the course of MS is largely unknown. The aim of our preliminary study was to analyse in monocytes of relapsing-remitting and progressive forms of MS patients the expression of a set of miRNAs which impact monocyte-macrophage immune function and their communication with brain cells. Quantitative PCR showed that miRNAs with anti-inflammatory functions, which promote pro-regenerative polarization, are increased in MS patients, while pro-inflammatory miR-155 is downregulated in the same patients. These changes may indicate the attempt of monocytes to counteract neuroinflammation. miR-124, an anti-inflammatory marker but also of myeloid cell quiescence was strongly downregulated, especially in progressive MS patients, suggesting complete loss of homeostatic monocyte function in the progressive disease phase. Profiling of miRNAs that control monocyte polarization may help to define not only the activation state of monocytes in the course of the disease but also novel pathogenic mechanisms.

## Introduction

Multiple Sclerosis (MS) is an autoimmune disease of the central nervous system (CNS) characterized by inflammatory demyelination and neurodegeneration that affects 2.5 million people worldwide^1,2^. The clinical forms of the disease are represented by the relapsing-remitting (RRMS) course, characterized by episodes of neurological dysfunction that may recover either completely or incompletely, and by the progressive course, either primary (PPMS) or secondary (SPMS), characterized by neurological dysfunction that intensify progressively^3^.

Etiologically, MS is a complex disorder in which genetic susceptibility, environmental factors, and epigenetic modifications play a role^4^.

It is widely accepted that activation of peripheral T cells is the main pathogenic event in MS although other components of the immune system are implicated^3^, such as infiltrating and brain resident microglia. Macrophages differentiate from circulating blood monocytes that mainly enter the CNS as part of an acute inflammatory response, and contribute to disease progression and demyelinating activity^5^. Monocytes-macrophages are the prevalent cell type encountered at myelin lesion sites^6^ and many of them are in close apposition to degenerating axons^7^. Depending on environmental stimuli, macrophages adopt a wide variety of phenotypes via undergoing different phenotypic polarization^8^. On the base of the inflammatory response, macrophages have been distinguished into two opposite states, pro-inflammatory and pro-regenerative, involved in the killing and removal of pathogens and in the resolution phase of inflammation, respectively^9^. The polarization of macrophages is defined by a set of markers, depending on surface receptor expression, effects or functions, cytokine and chemokine production^10^. For example, interleukin-1β (IL-1β), and TNFalpha (TNF-α) are typically associated with a pro-inflammatory state, whereas chitinase 3 like 1 (CHI3L1), and IL-10 are indicative of pro-regenerative phenotype^8^. In other cell types, however, these polarization markers may reflect a distinct activation state, with CHI3L1 being, for example, a marker of neuron degeneration and dysfunction^11^. Recently, in the experimental autoimmune encephalomyelitis (EAE) model of MS infiltrating phagocytes have been shown to acquire a pro-inflammatory polarization at the onset of disease and to later switch towards an anti-inflammatory state in response to CNS-derived signals^12^. Still, macrophage state evolution during the formation and resolution of myelin lesion in MS is largely unknown.

In the last decades, many studies have been carried out with the purpose of identifying MS biomarkers that can improve disease diagnosis, predict disease progression, and enhance clinical outcomes. The research of MS biomarkers has recently focused on microRNAs (miRNAs) in accessible body fluids. miRNAs are small single stranded non-coding RNAs which are known to regulate post-translational transcription^13^, by either targeting mRNA degradation or by stopping protein translation. miRNAs are very abundant in the brain and in the immune system, where they critically control monocyte-macrophage activation and polarization^14^. Since an individual miRNA is able to control many target genes, altered expression of singles miRNAs influences several biological processes in both immune and brain cells. Importantly, miRNAs are released by brain cells and circulate in human body fluids bound to RNA-processing molecules or packaged inside extracellular vesicles (EVs), where they may reflect alterations of the brain status, representing a brain “fluid biopsy”^15–17^.

miRNAs extracted from blood cells may provide numerous advantages as pathological markers of MS, being available with a minimally invasive method and providing at the same time information on the activation state of immune cells which enter the brain in the disease and therefore on the effectiveness of anti-inflammatory therapy.

In MS patients miRNAs were first profiled in blood-derived cells and in active lesions. Subsequently, miRNAs were analysed in the biological fluids, i.e. serum and cerebrospinal fluid (CSF)^18^. These studies led to the identification of a number of miRNAs as candidate MS biomarkers, including miR-223^18–21^, miR-146a^18,20,22^, miR-155^18,20,22,23^, miR-181a and b^18,24^, miR-23a^18,24,25^, miR-124^18^ and miR-30c^18,26^. All these miRNAs are involved in macrophages polarization^14,24,27–29^ with miR-124 being key driver of cell quiescence and pro-regenerative state^30^ and miR-155 of pro-inflammatory phenotype. Importantly, the miRNAs mentioned above may not only influence macrophage effector function but also control neuronal and/or oligodendrocyte activity, by silencing genes involved in synaptic transmission and myelin production^18,31–33^.

In light of these considerations in the preliminary study, we analysed by Real-time PCR this set of miRNAs in monocytes isolated from RRMS and PPMS subjects, followed by the assessment of the monocyte/macrophage polarization through the analysis of some phenotypic markers. Their expression levels provided some insights about the polarization state of monocytes in the two disease forms and their possible interactions with brain cells.

## Results

### miRNAs expression levels in monocytes

**Figure 1:**
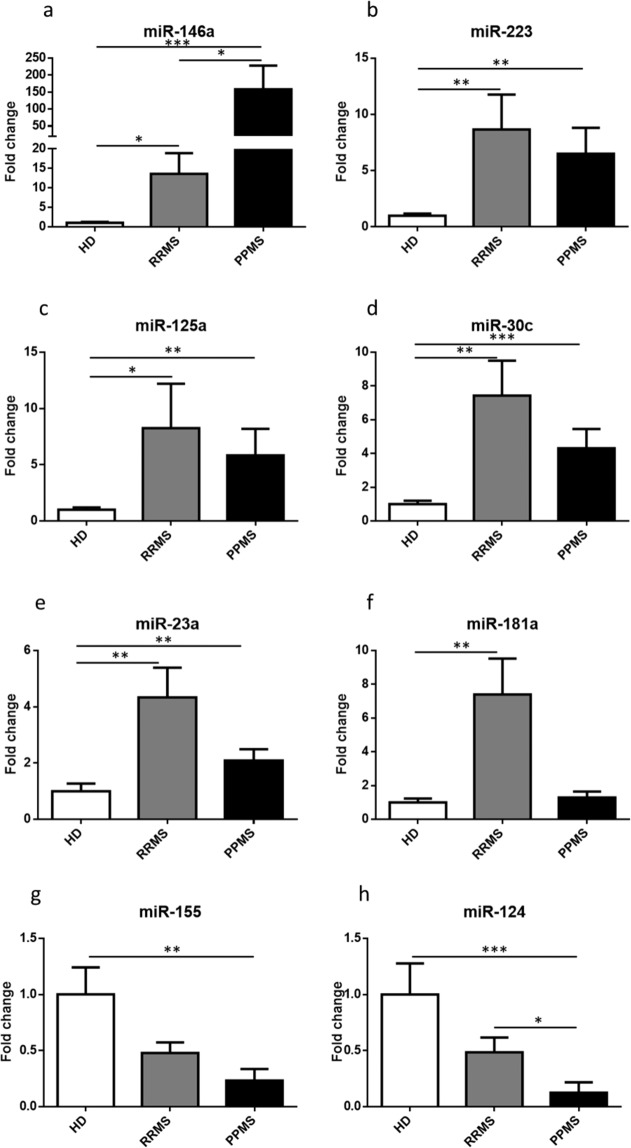
miRNAs expression levels in monocytes of HDs and MS patients by Real-Time PCR. Quantitative Real-Time PCR analysis of (**a**) mir-146a; (**b**) mir-223; (**c**) mir-125a; (**d**) mir-30c; (**e**) mir-181a; (**f**) mir-23a; (**g**) mir-155; (**h**) miR-124 was performed. The relative expression levels were calculated using the comparative Ct method, with RNU1A1 as endogenous control. Data are expressed as mean ± SEM of fold change values (*p < 0.05; **p < 0.01; ***p < 0.001), Kruskal-Wallis followed by Mann Whitney U test.

Results from qPCR analysis revealed dysregulation of all tested miRNAs in MS patients as compared to sex- and aged-matched healthy controls. Significance levels and fold differences were calculated for the RRMS patients, PPMS patients and controls; the criterion for differential expression of miRNA was set to P < 0,05. Specifically, increased expression levels of miR-146a (Fig. 1a), miR-223 (Fig. 1b), miR-125a (Fig. 1c) miR-30c (Fig. 1d), and miR-23a (Fig. 1e) were found in both RRMS and PPMS patients as compared to controls. miR-181a (Fig. 1f) was augmented in RRMS but not in PPMS patients compared to HDs. On the contrary, decreased expression of miR-155 (Fig. 1g) was observed in both PPMS and RRMS patients compared to HDs. Similarly, reduced levels of miR-124 (Fig. 1h) were found in PPMS patients compared to HDs and RRMS patients.

### Phenotypic mRNAs expression levels in monocytes

**Figure 2:**
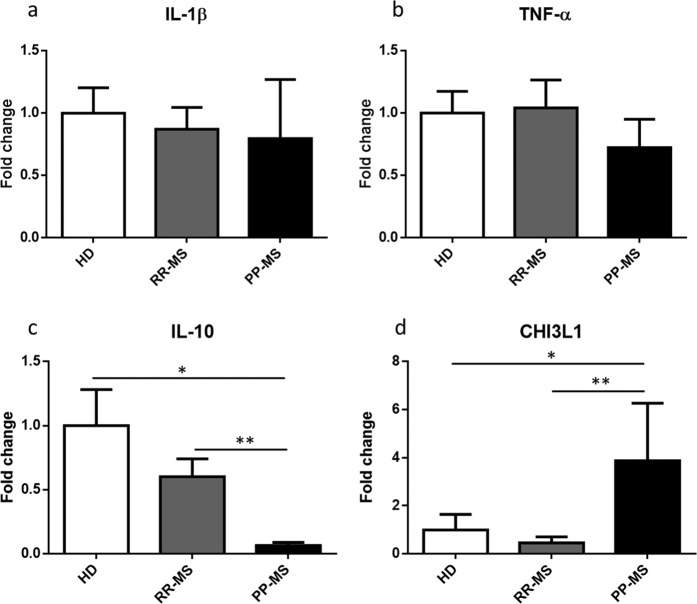
Phenotypic mRNAs expression levels in monocytes of HDs and MS patients by Real-Time PCR. Quantitative Real-Time PCR analysis of **(a**) IL-1β; (**b**) TNF-α; (**c**) IL-10; (**d**) CHI3L1 was performed. The relative expression levels were calculated using the comparative Ct method, with ACT-β as endogenous control. Data are expressed as mean ± SEM of fold change values (*p < 0.05; **p < 0.01; ***p < 0.001), Kruskal-Wallis followed by Mann Whitney U test.

To investigate macrophage polarization, gene expression of phenotypic markers was assessed. IL-1-β and TNF-α were used as markers of a pro-inflammatory state, whereas IL-10 and CHI3L1 were used as markers for pro-regenerative polarization. No expression changes were observed for IL-1-β between different groups (Fig. 2a). Likewise, no difference was observed for TNF-α (Fig. 2b), although a slight reduction was noticed in PPMS patients compared to HDs. Reduced levels of IL-10 were found in PPMS patients compared to HDs and RRMS patients (Fig. 2c). On the contrary, CHI3L1 augmented in PPMS patients compared to HDs and RRMS patients (Fig. 2d).

## Discussion

Monocytes/macrophages are thought to be the most predominant immune cell type responsible for cellular pathology and tissue damage in MS^34^. Previous studies provided insights into their activation state during MS evolution, though full functional characterization of their phenotype is still lacking^35,36^.

In this exploratory study, we analysed the expression of a set of miRNAs which control monocyte-macrophage polarization in a small cohort of MS patients in clinical remission. We found recognizable patterns of miRNAs expression which may help to define the monocytes/macrophages activation state in RRMS and PPMS patients, representing possible disease biomarkers. However, a limitation of our study is that the microRNAs and phenotypic markers were analysed only in patients in clinical remission and not with active disease. The inclusion of relapsing patients in future studies will be fundamental to strengthen the present findings and validate the miRNA expression patterns as disease biomarkers.

We show that miR-146a-5p is elevated in both RRMS and PPMS monocytes. The miRNA is a key player of innate immunity that acts as a brake on inflammation. miR-146a-5p is upregulated in inflamed immune cells but has anti-inflammatory activity by inhibiting NF-kB activity via two signal transducers: TNF receptor-associated factor 6 (TRAF6) and IL-1 receptor-associated kinase 1 (IRAK 1)^37^ resulting in reduction of TNF-α, IL-6 and IL-1β^38^. miR-146a also controls the expression of genes involved in energy metabolism. It reduces glycolytic activity and fatty acid synthesis, a metabolic change that may favour pro-regenerative polarization in monocytes-macrophages^39^. Importantly, miR-146a produced by reactive microglia has been recently shown to impair proper synaptic function and stability through silencing of both pre- and post-synaptic genes^32^. This suggests that miR-146a produced by monocytes-macrophages in the periphery may also regulate gene expression in the CNS grey matter, as previously proposed by Ridder and colleagues^40^.

Similarly, to miR-146a, miR-223 is an anti-inflammatory miRNA, highly expressed in myeloid cells that controls NF-kB^21^ and STAT5^25^ and promotes regenerative myeloid cell phenotype^41^. miR-223 expression is elevated in RRMS and, at lesser extent, in PPMS patients. This finding is in line with previous studies^20,21,41,42^, showing stronger miR-223 upregulation in PBMCs of RRMS compared to PPMS patients. In addition to anti-inflammatory function, miR-223 is required for activation of PPAR-ɣ, a key regulator of cell metabolism that can contribute to drive pro-regenerative polarization in monocyte/macrophages^43^. Accordingly, miR-223 released by monocyte/macrophages via EVs modulates monocyte/macrophage differentiation^44^.

miR-125a, upregulated in both RRMS and PPMS, is an additional miRNA that promotes pro-regenerative polarization in macrophages through downregulation of TNF-a-induced protein 3 (TNFAIP3) and of the inflammatory transcription factor Kruppel-like factor13 (KLF13)^45^ and KLF4^14^. The miRNA may also reduce monocyte migration through blood brain barrier cells and inflammatory cytokine secretion^46^. Interestingly, miR-125a also targets neuronal genes, including synaptotagmin II and the NMDA subunit GRIN2A as predicted by TargetScan database, potentially altering the interplay between macrophages-monocyte and neurons.

Finally, miR-181a^39,47^, selectively upregulated in RRMS, targets inflammatory molecules, i.e. IL1-α, MAPK1, TNF-α, and TLR4 and reduces macrophage polarization toward pro-inflammatory phenotype^24,28^.

Collectively, our data confirm elevated levels of miR-146a and miR-223 in the peripheral monocytes of a small cohort of MS patients and show high expression of two additional miRNAs with anti-inflammatory functions, i.e. miR-125a and miR-181a in RRMS. Only miR-125a is overexpressed along with miR146a and miR-223 in PPMS. Upregulation of anti-inflammatory/pro-regenerative miRNAs might be considered a counter-active mechanism to dampen an inflammatory reaction during remitting and the progressive phase of MS.

Little is known about the role of miR-30c in the inflammatory response. Recently, Ceolotto *et al*. investigated the role of miR-30c-5p in atherosclerosis pathways, and suggested an anti-inflammatory action also for this miRNA^29^, as evidenced by IL-1β production under miR-30c downregulation^29^. Thus, elevated levels of miR-30c may contribute to dampen the inflammatory response mostly in RRMS, where the miRNA is significantly altered.

miR-23a is more expressed in pro-inflammatory versus pro-regenerative macrophages^27^ and overexpressed in inflammatory microglia^32^. It targets many neuronal genes, as predicted by TargetScan database including FGF-2, a growth factor involved in nervous system development^21^. Elevated FGF-2 levels were previously reported in the CSF of MS patients, particularly those with active disease^48^. Elevated miR-23a levels were found in RRMS patients^24^, with a normalization of the miRNA levels upon Fingolimod treatment^25^. We here confirmed miR-23a overexpression in monocytes from RRMS patients, supporting its role as a pro-inflammatory monocyte marker. Further analysis will be required to explore whether the miRNA may also represent a marker of neuron degeneration in MS.

Among miRNAs clearly recognized in MS pathogenesis, miR-155 is the most studied for its role in both immune and brain cells, and it is an established marker of inflammatory monocytes-macrophages. Its expression is elevated in both circulating CD14+ monocytes and at active lesions^20,23^.

Interestingly miR-155 targets many fundamental neuronal genes (shank-2, GRIN2A, BDNF), as predicted by TargetScan database^49^ but also genes involved in oligodendrocyte differentiation, potentially affecting communication between monocyte-macrophages, neurons and/or oligodendrocytes^50^. Low expression of this pro-inflammatory miRNA in PPMS and RRMS monocytes compared to HDs is consistent with the upregulation of many anti-inflammatory miRNAs in the same subjects.

Finally, miR-124 is the first miRNA that was linked to pro-regenerative macrophage polarization^14^. Its expression can be induced in monocytes and macrophages in response to the Th2 cytokines^51^. However, *in vivo* the miRNA is mainly expressed in microglia, where it promotes cell quiescence and homeostatic functions^52^. Thus we speculate that in analogy to microglia, reduced levels of miR-124 in RRMS but especially PPMS monocytes may reflect loss of homeostatic function and inability of the cells to shift towards pro-regenerative state.

Interestingly miR-155 and miR-124 are more strongly downregulated in PPMS compared to RRMS while anti-inflammatory miRNAs are more abundant in RRMS compared to PPMS, suggesting that monocytes undergo distinct phenotypic changes in the two forms of MS.

To get further insights into the activation state of monocytes in PPMS and RRMS we analysed the expression of widely used polarization markers, i.e. IL-1β, TNF-α, IL-10 and CHI3L1. No variations in IL-1β expression were observed in RRMS and PPMS patients compared to HDs, excluding major alterations in the pro-inflammatory state of the cells. Similarly, TNF-α expression did not change significantly between different groups, although a trend to decrease was noted in PPMS patients. This reduction correlates with the up-regulation of miR-146a-5p observed in PPMS subjects, suggesting that the miRNA influences the expression of its own target, as mentioned above.

TNF-α is a pleiotropic cytokine that often shows contradictory effects, particularly in the CNS, in which can either promote or impair myelinating processes. It was reported that TNF-α may favour proliferation of immature oligodendrocytes and myelin repair via TNFR2 receptors, therefore its reduction in PPMS could contribute to remyelination failure^53^.

IL-10 is a powerful anti-inflammatory cytokine that downregulates the immune responses and subsequent tissue immunopathology^54^. Its higher expression in RRMS patients compared to PPMS, albeit lower than in controls, may be in line with the attempt of monocytes to establish an anti-inflammatory response at early disease stages. On the other hand, the downregulation of this cytokine in PPMS patients along with TNF-α reduction could explain exacerbation of demyelination processes in these patients.

An opposite trend was observed for the expression of CHI3L1, that is indeed, emerging as a potential marker of disease activity in MS^55–57^. Our results are in line with the work by Burman and colleagues, who showed increased levels of CHI3L1 in SPMS patients, highlighting a correlation of CHI3L1 expression with tissue damage and disability^56^. Furthermore, CHI3L1 expression was previously associated with destruction of the extracellular matrix and tissue remodelling^58^ that could be responsible for neuronal dysfunction^11^. Therefore, the decrease in CHI3L1 expression in RRMS may once again reflect the attempt of monocytes to acquire a pro-regenerative phenotype.

In conclusion, our results show that miRNAs with anti-inflammatory functions, which promote pro-regenerative polarization, are increased in MS patients, while miR-155, the prototypical pro-inflammatory miRNA is downregulated in the same patients. These changes may reflect the attempt of monocytes to establish an anti-inflammatory/pro-regenerative response in MS. However, miR-124, a marker of quiescence and anti-inflammatory polarization in myeloid state, is also strongly downregulated, especially in patients with progressive MS, suggesting persistent monocyte activation during disease progression. In line with this hypothesis, assessment of phenotypic monocyte-macrophage markers indicates a decrease in TNF-α and IL-10, which may favour remyelination, and elevated expression of CHI3L1, an emerging marker of MS activity in monocytes from PPMS.

However, profiling of miRNAs that impact monocyte immune function and their communication with brain cells is the first step towards more extensive characterization of monocyte-macrophage polarization in patients with relapsing remitting or progressive MS.

Further studies on larger cohorts of MS patients are needed to assess (i) the evolution of monocytes/macrophages phenotype in the course of MS, especially during transition to the progressive phase of the disease, (ii) their polarization in active MS patients and, (iii) to explore how monocyte polarization impacts the interplay of monocytes with brain cells. Understanding how monocyte polarization evolves during MS could not only provide novel biomarkers to monitor the disease but it could also provide novel therapeutic opportunities.

## Materials and Methods

### Patients and controls

Twenty-one RRMS patients (6M/15F, mean age 38 ± 9, EDSS 2.9 ± 1.4) and 8 PPMS patients (1M/7F, mean age 47 ± 11, EDSS 5.9 ± 1.3) were recruited at the MS Center, Neurology Unit, Department of Medical and Surgical Sciences, University of Foggia and the MS Center of the San Raffaele Hospital of Milan. Sixteen healthy donors (HDs) (10M/6F, mean age 45 ± 11) were similarly investigated. All patients were clinically not active at the time of blood withdrawal, therefore totally steroid free, with MRI comparable to that of the previous year, without T2 and Gd+ lesions^59^. Venous blood sample (20 ml each) were collected in EDTA tubes and processed within one hour postcollection in order to obtain purified monocytes from total peripheral blood mononuclear cells (PBMCs). Patients had received no disease modifying drugs during the past 3 months. Criteria for volunteer selection consist of no recent illness or treatment for a chronic medical condition. No medical history was obtained from donors. The study has been reviewed and approved by the Ethical Committe of Ospedali Riuniti Foggia/University of Foggia and San Raffaele Hospital of Milan. All subjects gave written informed consent to sign before blood samples were collected. All investigations conformed to the principles outlined in the Declaration of Helsinki.

### Preparation of monocytes

PBMCs from patients with MS and HDs were isolated from freshly drawn venous blood by density centrifugation, using a Lymphosep, Lymphocyte separation Media (d = 1,077 g/ml) gradient (Biowest) as described by the manufacturer. Mononuclear cell suspension was prepared at a concentration of 1 × 10^8^ cells/ml in recommended medium containing PBS 1×, 2%FBS, and 1 mM EDTA. Monocytes were isolated according to the previous reported method^60^. Briefly, monocyte positive selection by monoclonal CD14 antibody-conjugated microbeads was performed by using EasySep Human CD14 Selection kit (Stemcell Technologies) according to the manufacturer’s protocol, after the cells were resuspended in RPMI 1640 medium (Sigma) enriched with FBS at 10% (Gibco Invitrogen). 10 µl of the positively selected cells was mixed with 10 µl of 4% trypan blue solution and live/dead cells were counted in a Bio-Rad TC-10 Automated Cell Counter in order to plate 1 × 10^6^ cells into 35 mm dishes and they were then incubated at 37 °C and 5% CO_2_ for 2 hours. After extensive washing, monocytes were exposed for 30 min to 200 µM Benzoyl- ATP dissolved in 1 mL of Krebs-Ringer solution (KRH, 125 mM NaCl, 5 mM KCl, 1.2 mM MgSO_4_, 1.2 mM KH_2_PO, 2 mM CaCl_2_, 6 mM D-glucose, and 25 mM HEPES/NaOH, pH 7.4) at 37 °C.

### Total RNA isolation and quantitative Real-Time PCR analysis

In order to perform miRNA analysis, total RNA was isolated from monocytes using Direct-zol RNA MiniPrep kit (Zymo Research), according to the manufacturer’s instructions. Concentration of RNA was determined by measuring the absorbance at 260 nm with a NanoDrop 1000 Spectrophotometer. RNA for miRNAs analysis (10 ng) was reverse transcribed into cDNA using Universal cDNA synthesis kit II (Exiqon). The resulting cDNA transcript were used for PCR amplification using ExiLENT SYBR Green Master Mix II (Exiqon) and miRNA specific primer set (miRCURY LNA Universal RT microRNA PCR, Exiqon) for miR-146a-5p, miR-181a-5p, miR-223-3p, miR-23a-3p, miR-30c-5p, miR-125a-5p, miR-155-5p and miR-124-3p (Table 1).

**Table 1:** Primers used for miRNAs/mRNA analysis.

Primers	Source	Cat.numbers
hsa-miR-146a-5p	Exiqon	204688
hsa-miR-181a-5p	Exiqon	206081
hsa-miR-223-3p	Exiqon	205986
hsa-miR-23a-3p	Exiqon	204772
hsa-miR-30c-5p	Exiqon	204783
hsa-miR-125a-5p	Exiqon	204339
hsa-miR-155a-5p	Exiqon	204308
hsa-miR-124a-3p	Exiqon	206026
hsa-RNU1A1	Exiqon	203909
IL-1-β	Applied Biosystems	Hs00174097_m1
TNF-α	Applied Biosystems	Hs01113624_g1
CHI3L1	Applied Biosystems	Hs00609691_m1
IL10	Applied Biosystems	Hs00961622_m1
ACT-β	Applied Biosystems	Hs01060665_g1

RNA for M1/M2 marker analysis (100 ng) was reverse transcribed into cDNA using High Capacity cDNA Reverse Transcription Kits (Applied Biosystem). The resulting cDNA transcript were used for PCR amplification using TaqMan Universal Master Mix II (Applied Biosystems) and gene specific Taqman assays (Applied Biosystems) for IL-1β, TNF-α, CHI3L1, IL-10 (Table 1).

Quantitative real-time PCR was performed with a Step One Real time PCR system (Applied Biosystems). The relative expression levels were calculated using the comparative Ct method, with RNU1A1 and beta-actin (ACT-β) as endogenous control.

### Statistical analysis

Statistical comparison of miRNAs and mRNA expression between different groups was performed using the multiple comparison non-parametric Kruskal-Wallis followed by the non-parametric Mann Whitney U test as post-hoc test. P value <0.05 was considered significant. All analyses were performed using GraphPad Prism version 5.0.
